# Biologically informed deep neural network for prostate cancer discovery

**DOI:** 10.1038/s41586-021-03922-4

**Published:** 2021-09-22

**Authors:** Haitham A. Elmarakeby, Justin Hwang, Rand Arafeh, Jett Crowdis, Sydney Gang, David Liu, Saud H. AlDubayan, Keyan Salari, Steven Kregel, Camden Richter, Taylor E. Arnoff, Jihye Park, William C. Hahn, Eliezer M. Van Allen

**Affiliations:** 1 Dana-Farber Cancer Institute, Boston, MA USA; 2 Broad Institute of MIT and Harvard, Cambridge, MA USA; 3 Al-Azhar University, Cairo, Egypt; 4 University of Minnesota, Division of Hematology, Oncology and Transplantation, Minneapolis, MN USA; 5 Department of Urology, Massachusetts General Hospital, Harvard Medical School, Boston, MA USA; 6 Department of Pathology, University of Illinois at Chicago, Chicago, IL USA

**Keywords:** Cancer genomics, Computer science

## Abstract

The determination of molecular features that mediate clinically aggressive phenotypes in prostate cancer remains a major biological and clinical challenge^1,2^. Recent advances in interpretability of machine learning models as applied to biomedical problems may enable discovery and prediction in clinical cancer genomics^3–5^. Here we developed P-NET—a biologically informed deep learning model—to stratify patients with prostate cancer by treatment-resistance state and evaluate molecular drivers of treatment resistance for therapeutic targeting through complete model interpretability. We demonstrate that P-NET can predict cancer state using molecular data with a performance that is superior to other modelling approaches. Moreover, the biological interpretability within P-NET revealed established and novel molecularly altered candidates, such as *MDM4* and *FGFR1*, which were implicated in predicting advanced disease and validated in vitro. Broadly, biologically informed fully interpretable neural networks enable preclinical discovery and clinical prediction in prostate cancer and may have general applicability across cancer types.

## Abstract (web summary)

A biologically informed, interpretable deep learning model has been developed to evaluate molecular drivers of resistance to cancer treatment, predict clinical outcomes and guide hypotheses on disease progression.

## Main

With the advancement of molecular profiling technologies, the ability to observe millions of genomic, transcriptional and additional features from patients with cancer and their tumours has grown markedly over the past decade. Specifically, in prostate cancer, the availability of rich molecular profiling data linked to clinical annotation has enabled discovery of many individual genes, pathways, and complexes that promote lethal castration-resistant prostate cancer (CRPC), which has led to both biological investigations and clinical evaluations of these individual features for predictive utility^1,2,6–12^. However, the relationships between these molecular features and their combined predictive and biological contributions to disease progression, drug resistance and lethal outcomes remain largely uncharacterized.

There is a wide range of potential approaches when developing a predictive model, although each comes with trade-offs of accuracy and interpretability. In translational cancer genomics, interpretability of predictive models is critical, as properties that contribute to the predictive capabilities of the model may not only inform patient care, but also provide insights into the underlying biological processes to prompt functional investigation and therapeutic targeting. Linear models such as logistic regression tend to have high interpretability with less accurate predictive performance, whereas deep learning models often have less interpretability but higher predictive performance^13,14^. Using a typical fully connected dense deep learning approach for building predictive models may also result in overfitting unless the network is well regularized, and such models have a tendency to be computationally expensive and less interpretable^15^.

Efforts to search for slimmer architecture and sparse networks given a full model demonstrated that sparse models can decrease storage requirements and improve computational performance^16–18^. However, finding such a sparse model may be challenging, since the typical training–pruning–retraining cycle is usually computationally expensive, and recent studies indicate that building a sparse model de novo may be easier^19^. In addition, efforts to enhance the interpretability of deep learning models and the need to explain model decisions led to the development of multiple attribution methods, including LIME^20^, DeepLIFT^13^, DeepExplain^21^ and SHAP^22^, that can be used to enhance the deep learning explainability and understand how the model is processing information and making decisions.

Together, the advances in sparse model development and attribution methods have informed the development of deep learning models to solve biological problems using customized neural network architectures that are inspired by biological systems. For example, visible neural networks were developed to model the effect of gene interaction on cell growth in yeast (DCell) and cancer cell line interactions with therapies (DrugCell)^3,5^. A pathway-associated sparse deep neural network (PASNet) used a flattened version of pathways to predict patient prognosis in Glioblastoma multiforme^23^. However, whether biologically informed neural networks can accelerate biological discovery with translational potential and simultaneously enable clinical predictive modelling is largely unknown. Here we hypothesized that a biologically informed deep learning model built on advances in sparse deep learning architectures, encoding of biological information and incorporation of explainability algorithms would achieve superior predictive performance compared with established models and reveal novel patterns of treatment resistance in prostate cancer, with translational implications.

## Results

**Fig. 1:**
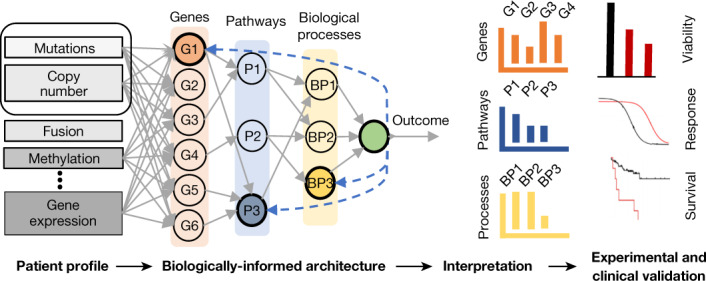
Interpretable biologically informed deep learning. P-NET is a neural network architecture that encodes different biological entities into a neural network language with customized connections between consecutive layers (that is, features from patient profile, genes, pathways, biological processes and outcome). In this study, we focus primarily on processing mutations and copy-number alterations. The trained P-NET provides a relative ranking of nodes in each layer to inform generation of biological hypotheses. Solid lines show the flow of information from the inputs to generate the outcome and dashed lines show the direction of calculating the importance score of different nodes. Candidate genes are validated to understand their function and mechanism of action.

**Extended Data Fig. 1:**
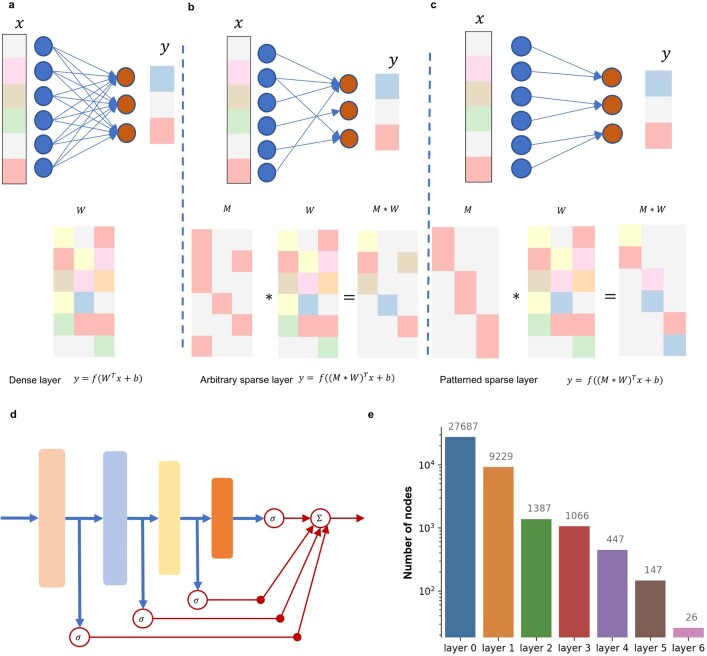
P-NET architecture and characteristics. **a**, Dense layer with inputs $x\in {R}^{{dx}}$ and output $y\in {R}^{{dy}}$ vectors. The matrix $W\in {R}^{{dx}\ast {dy}}$ is a trainable weights matrix and $b\in {R}^{{dy}}$ is the bias vector. $f$ is the layer activation function. **b**, Arbitrary sparse layers are flexible to encode any connection scheme with the added $M\in {\{\mathrm{0,1}\}}^{{dx}\ast {dy}}$ binary mask matrix that controls the connectivity of the layer imposing sparsity on the weights matrix. **c**, A patterned sparse matrix with mask matrix $M$ that follows a certain pattern. This pattern can be used to make computations more efficient. **d**, Predictive node is connected to each hidden layer in P-NET, and the final prediction is calculated by taking the average of all the predictive elements in the network. **e**, The number of parameters per layer of P-NET.

We developed a deep-learning predictive model that incorporates previous biologically established hierarchical knowledge in a neural network language to predict cancer state in patients with prostate cancer on the basis of their genomic profiles. A set of 3,007 curated biological pathways were used to build a pathway-aware multi-layered hierarchical network (P-NET) (Methods). In P-NET, the molecular profile of the individual is fed into the model and distributed over a layer of nodes representing a set of genes using weighted links (Fig. 1, Extended Data Fig. 1). Later layers of the network encode a set of pathways with increasing levels of abstraction, whereby lower layers represent fine pathways and later layers represent more complex biological pathways and biological processes. The connections between different layers are constrained to follow known child–parent relationships among encoded features, genes and pathways, and as a result the network is geared toward interpretability by design.

**Fig. 2:**
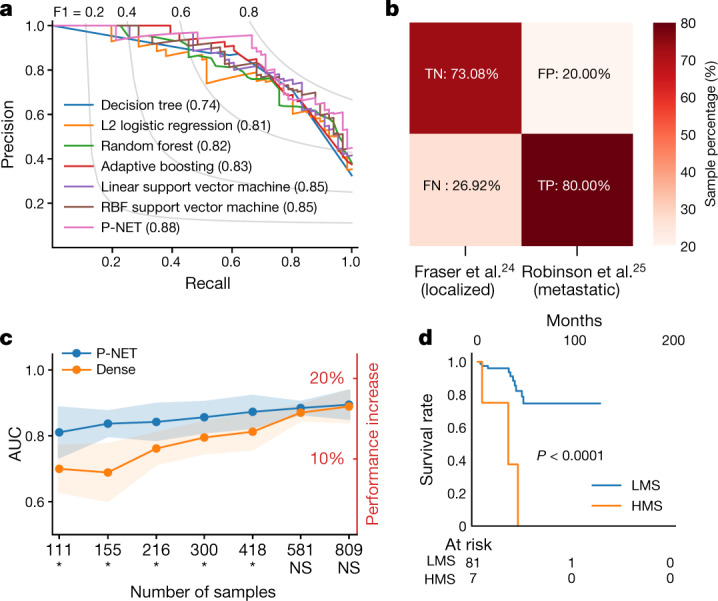
Prediction performance of P-NET. **a**, P-NET outperforms other models in terms of the AUPRC, values shown in brackets, when tested on the testing set (*n* = 204 from the Armenia et al. dataset^8^). RBF, radial basis function. **b**, When evaluated using two independent external validation cohorts^24,25^, P-NET achieves 73% true-negative rate (TN) and 80% true-positive rate (TP), showing that it can generalize to classify unseen samples. FN, false-negative rate; FP, false-positive rate. **c**, P-NET achieves better performance (measured as the average AUC over five cross-validation splits) with smaller numbers of samples compared to a dense fully connected network with the same number of parameters. The solid line represents the mean AUC and the bands represent mean ± s.d. (*n* = 5 experiments). The difference in performance is statistically significant for all sample sizes up to 500 (*P < 0.05, one-sided *t*-test) (Methods). **d**, Patients with primary prostate cancer and high P-NET scores, HPS,  (wrongly classified by P-NET to be resistant samples) have a greater tendency to exhibit biochemical recurrence (BCR) compared with patients with lower P-NET scores, LPS,  who tend to exhibit progression-free survival (*P* = 8 × 10^−5^; log-rank test, two sided). This shows that the P-NET model may be useful in stratifying patients in the clinic and predicting potential BCR (raw data are included in Supplementary Table 9). LPS, low P-NET score; HPS, high P-NET score.

**Extended Data Fig. 2:**
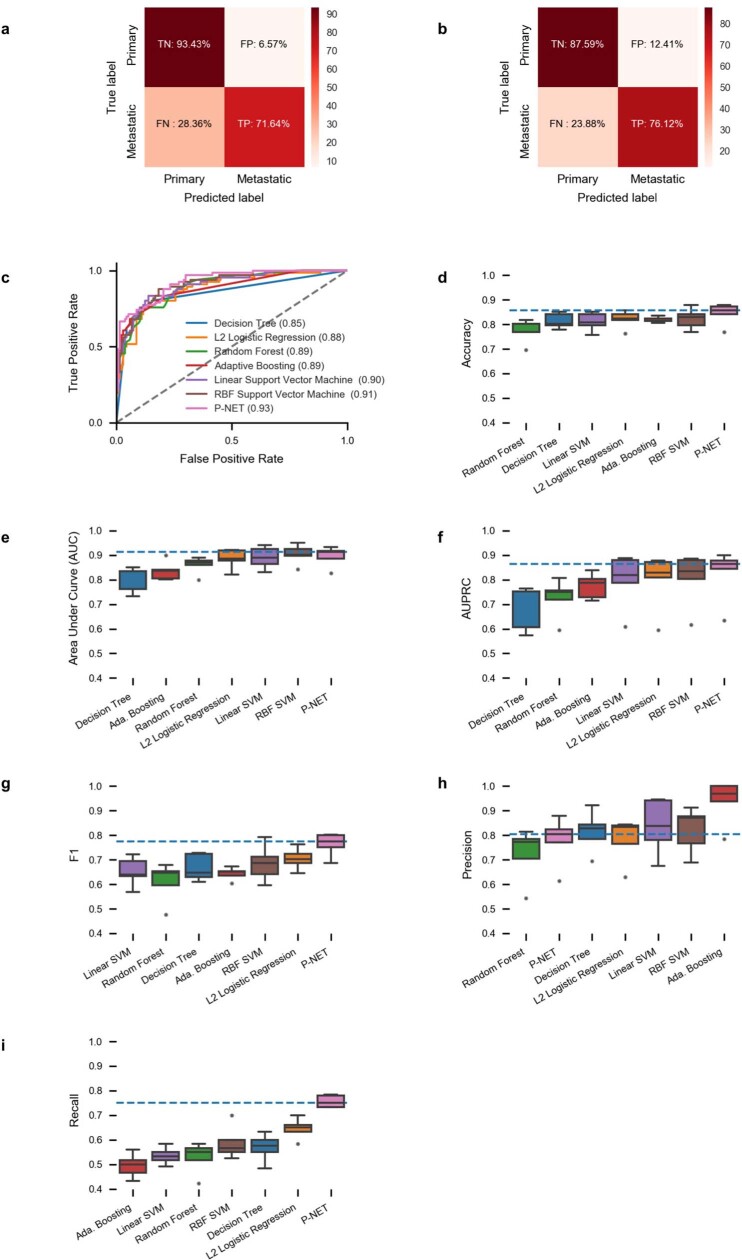
Computational performance of P-NET as compared to other models. **a**, Original confusion matrix calculated by using a typical 0.5 threshold of the prediction scores to generate binary predictions. **b**, Adjusted confusion matrix calculated using an adaptive threshold that is used to maximize F1 score. **c**, The ROC curve of P-NET compared to other models showing that P-NET outperforms other models in terms of the area under curve (AUC) when tested on the testing set (*n*=204 of Armenia et al dataset). The models are compared by repeatedly training and testing each model in a cross-validation setup (*n* = 5 experiments) with testing sample sizes of 188, 182, 182, 182, and 181 respectively. Performance metrics reported here include; accuracy (**d**), area under ROC curve (**e**), area under precision recall curve AUPRC (**f**), F1 measure (**g**), precision (**h**), and recall (**i**). P-NET outperforms other models on average using all the metrics except Precision. Data in **d-i** are represented as boxplots where the middle line is the median, the lower and upper hinges correspond to the 1st and 3rd quartiles, the whisker corresponds to the minimum or maximum values no further than 1.5 × IQR from the hinge (where IQR is the inter-quartile range). Data beyond the end of the whiskers are outlying points that are plotted individually.

**Extended Data Fig. 3:**
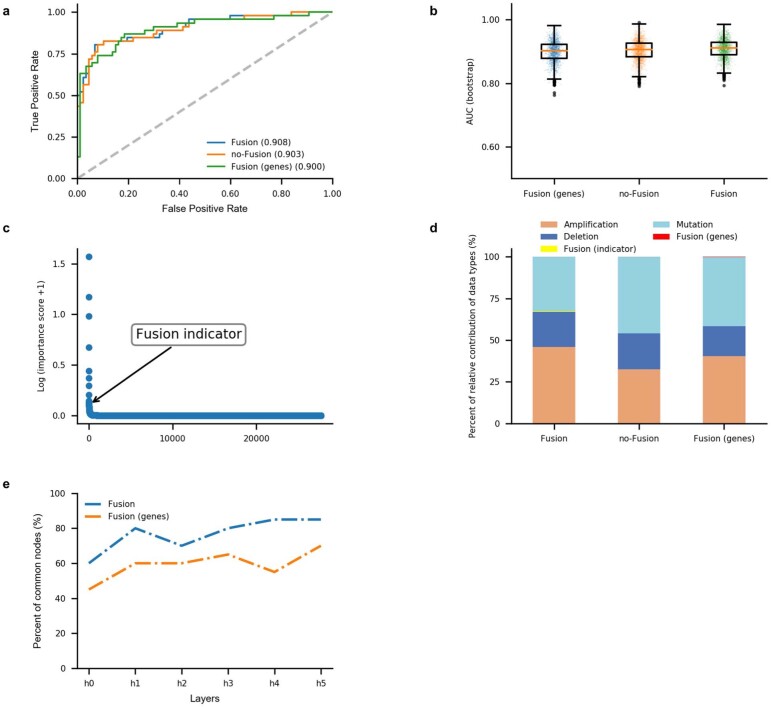
The effects of incorporating fusions in the P-NET model training. The effect of incorporating fusions in the P-NET model training. Three models are reported here to study the effect of fusion on the P-NET performance i) ‘no-Fusion’ model incorporating only copy number and mutations for each gene ii) ‘Fusion’ model where fusion is added to the model as one binary variable to indicate whether a certain sample has fusion or not (restricted to ETS fusions and oncogene fusions). ‘Fusion (genes)’ model where fusions are included as binary variables for each gene indicating whether a certain gene was involved in a fusion or not (restricted to ETS fusions and oncogene fusions). **a**, The AUC curve of the three trained models showing similar performance when tested on the testing set. **b**, A bootstrapped version of the AUC comparison (2000 bootstrap samplings) showing similar performance of the three models. **c**, The importance score of all features showing that the fusion indicator has a non-zero score even when it is added to the 27k features fed into the model. **d**) The overall contributions of different data types (calculated as the aggregation of the importance scores of all corresponding features) showing minor contributions of the fusion features. The signal from the fusion features goes smaller when distributed over genes (‘Fusions (genes)’ model) compared to the single feature encoding (‘Fusion’ model). **e**, The effect of adding fusion on the top ranked nodes in each layer as compared to the baseline ‘no-Fusion’ model rankings. Adding the fusion has a small effect on the top ranked nodes in higher layers, e.g. more than %80 of the top ranked nodes in h5 has not been affected by the fusion addition (‘Fusion’ model) compared to the baseline ‘no-Fusion’ model. The effect of the fusion addition is more prominent in the earlier layers, especially h0.

**Extended Data Fig. 4:**
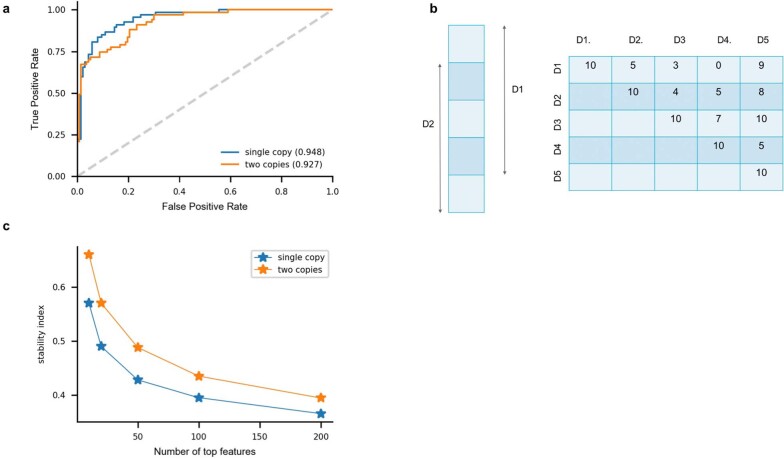
The effects of CNV definition on the P-NET model performance and stability. Two different models are trained on (i) mutations plus only high amplifications and deep deletions, referred to by ‘two copies’ in the legend and (ii) mutations plus all GISTIC2.0 states (deep deletion, deletion, neutral, amplification, high amplification) referred to by ‘single copy’ in the legends. **a**) AUC comparison between the two models showing slight increase in the performance when including all the copy number levels. **b**) The stability of top features is studied by comparing the overlap between features picked by the model over 5-fold data splits. The stability index is calculated for five data splits (D1-D5) where the cells show the overlap between top 10 features picked by the model for each pair of the data splits. **c**) comparing the stability index of the two models shows that restricting the copy number levels (‘two copies’ model) has a positive effect on stabilizing the features picked by the model when trained on different data splits.

**Extended Data Fig. 5:**
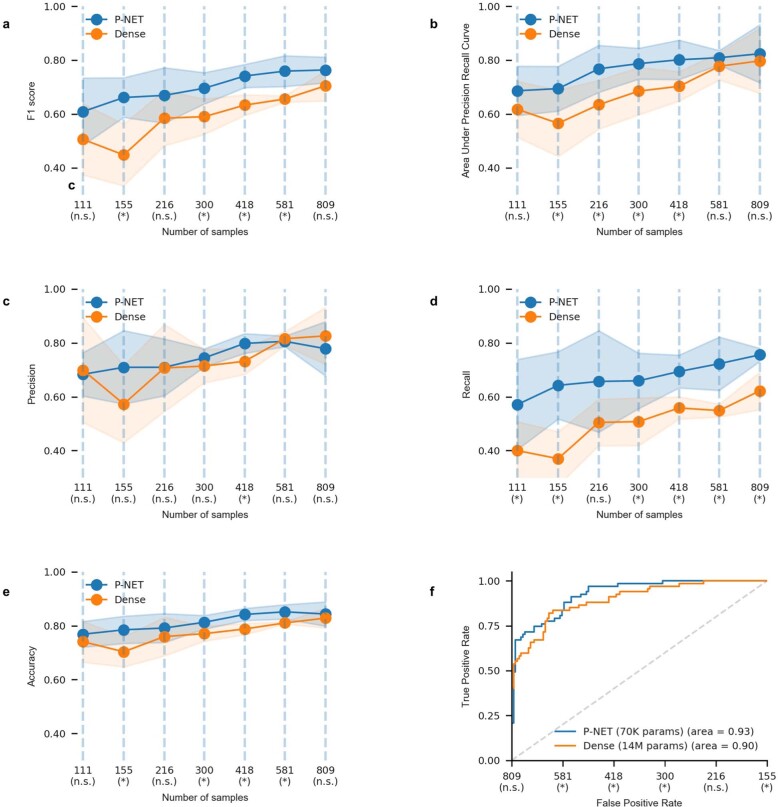
Performance comparison of sparse P-NET to dense models. Comparing the performance of P-NET to a dense network with the same number of trainable parameters using different sizes of training sets (**a**: Recall, **b**: Precision, **c**: AUPRC, **d**: F1, **e**: Accuracy). Sample sizes marked by (*) indicate statistically significant differences (*p*-value <0.05, one-sided *t*-test) while those marked by (n.s.) are not. The solid line represents the mean and the bands represent mean +/- SD (*n* =5 experiments). **f**, Comparison of P-NET to a dense model with the same architecture (same number of nodes) but with large number of trainable parameters (14 M) shows that sparse P-NET is still better than a dense model in terms of the area under ROC curve, AUC.

We trained and tested P-NET with a set of 1,013 prostate cancers (333 CRPCs and 680 primary cancers) (Methods; Supplementary Tables 1–5), divided into 80% training, 10% validation and 10% testing, to predict disease state (primary or metastatic disease) using somatic mutation and copy number data (Methods). The trained P-NET outperformed typical machine learning models, including linear and radial basis function support vector machine, logistic regression, and decision trees (area under the receiver operating characteristic (ROC) curve (AUC) = 0.93, area under the precision-recall curve (AUPRC) = 0.88, accuracy = 0.83) (Fig. 2, Extended Data Fig. 2, Supplementary Tables 6, 7, Methods). Incorporation of additional molecular features was feasible in P-NET (for example, fusions) but did not impact the performance of the model in this specific prediction task (Extended Data Figs. 3, 4). Furthermore, we evaluated whether the sparse model had characteristics distinct from a dense fully connected deep learning model. We trained a dense model with the same number of parameters as in the P-NET model on training sets with a logarithmically increasing number of samples from 100 to 811 (80% of the total number of samples). The mean performance (determined by AUC) of the P-NET model was higher than the dense model over all sample sizes, and this difference was statistically significant in smaller sample sizes (up to 500) (for example, mean AUC of fivefold cross-validation was significantly higher for P-NET compared with a dense network trained on 155 samples, *P* = 0.004) (Fig. 2c, Extended Data Fig. 5a–e; statistical test results are listed in Supplementary Table 8). Furthermore, a dense network that had the same number of neurons and layers as P-NET but a much larger number of parameters (14 million) also achieved inferior performance (Extended Data Fig. 5f).

We next performed external validation of the predictive aspects of the model using two additional prostate cancer validation cohorts, one primary^24^ and one metastatic^25^ (sample identifiers are listed in Supplementary Tables 4, 5; Methods). The trained P-NET model correctly classified 73% of the primary tumours and 80% of the metastatic tumours, indicating that the model can generalize to unseen samples with an adequate predictive performance (Fig. 2b). We hypothesized that patients with primary tumour samples incorrectly classified by P-NET as castration-resistant metastatic tumours may in fact have worse clinical outcomes. Patients with high P-NET scores misclassified as resistant disease were significantly more likely to have biochemical recurrence than patients with low P-NET scores (*P* = 8 × 10^−5^; log-rank test), indicating that for patients with primary prostate cancer, the P-NET score may be used to predict potential biochemical recurrence (Fig. 2d, Supplementary Table 9).

**Fig. 3:**
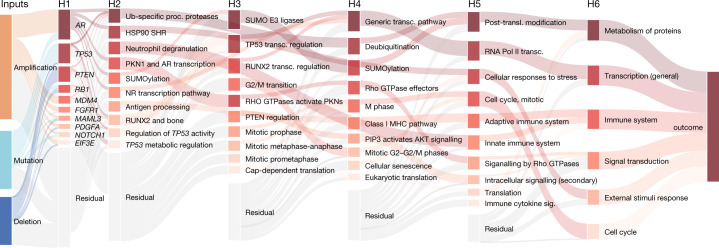
Inspecting and interpreting P-NET. Visualization of inner layers of P-NET shows the estimated relative importance of different nodes in each layer. Nodes on the far left represent feature types; the nodes in the second layer represent genes; the next layers represent higher-level biological entities; and the final layer represents the model outcome. Nodes with darker colours are more important, while transparent nodes represent the residual importance of undisplayed nodes in each layer. The contribution of a certain data type to the importance of each gene is depicted using the Sankey diagram—for example, the importance of the *AR* gene is driven mainly by gene amplification, the importance of *TP53* is driven by mutation, and the importance of *PTEN* is driven by deletion. NR, nuclear receptor; SHR, steroid hormone receptors; transc., transcription; transl., translation.

To understand the interactions between different features, genes, pathways and biological processes that contributed to the predictive performance and to study the paths of impact from the input to the outcome, we visualized the whole structure of P-NET with the fully interpretable layers after training (Fig. 3). Among aggregate molecular alterations, copy number variation was more informative compared with mutations, consistent with previous reports^26^. In addition, P-NET selected a hierarchy of pathways (out of 3,007 pathways on which P-NET was trained) as relevant to classification, including cell cycle checkpoints, post-translational modification (including ubiquitination and SUMOylation) and transcriptional regulation by *RUNX2* and *TP53*. Multiple members of the cell cycle pathway have been functionally implicated in metastatic prostate cancer, and specifically functionally interrogated in treatment-resistant contexts^27,28^. Ubiquitination and SUMOylation pathways contribute to the regulation of multiple tumour suppressors and oncogenes, including *AR*^29^, and dysregulation of these pathways has been linked to prostate cancer initiation and progression in preclinical models^30^. *RUNX2* is an osteogenic transcription factor that regulates cell proliferation and is associated with metastatic disease in patients with prostate cancer^31^.

**Extended Data Fig. 6:**
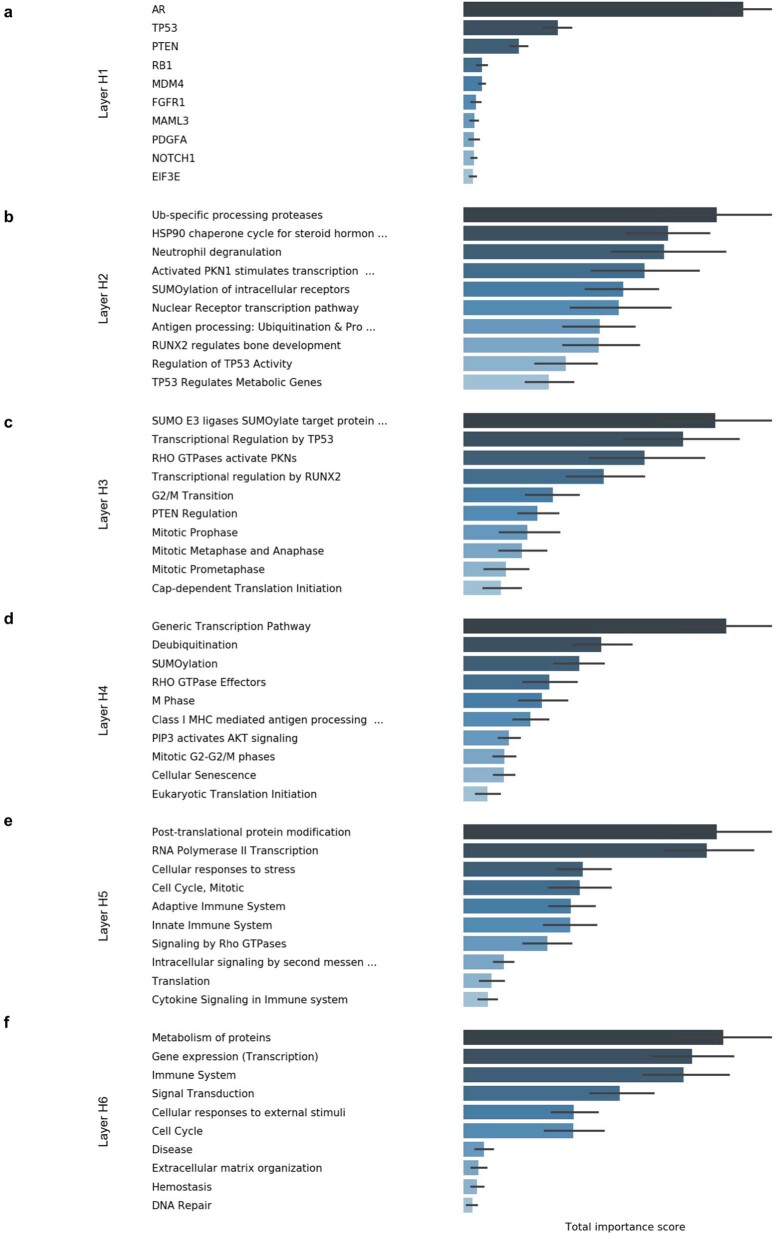
Relative ranking of nodes in each layer. Relative ranking of nodes in each layer based on P-NET total importance score. The height of the bar represents the estimated total importance score calculated as the summation of all sample-level importance scores over the testing set (*n* = 204). The error bar represents the 95-confidence interval around the estimated score calculated using 1000 bootstrap cycles over the testing set.

**Extended Data Fig. 7:**
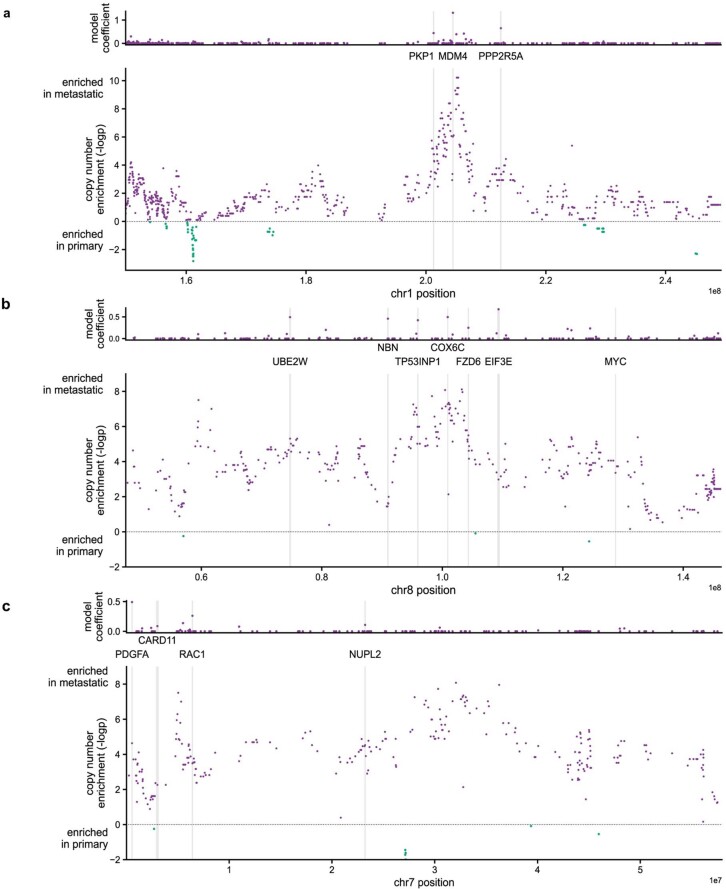
Relationship between P-NET importance scores and copy number enrichment of important genes. **a**, Copy number enrichment of genes on chr1p (containing *MDM4*) relative to their model importance score. The y-axis shows the enrichment of the amplification in metastatic samples relative to primary samples, using -log (signed p) from Fisher’s exact test. There is evidence of high amplification enrichment around *MDM4* specifically, but the higher model coefficient (importance score) is also partially informed by its relevance in biological pathways relative to neighboring genes (e.g. *PKP1*). **b**) There is less evidence for copy number focality being enriched around *EIF3E* on chr8q, which suggests that the model coefficient may be largely driven by the biological “bias” and less so by copy number focality. **c**, *PDGFA* on chr7p is a representative example where there is a mix of signal between modest focality at the peak where *PDGFA* is observed and biological “bias”.

**Extended Data Fig. 8:**
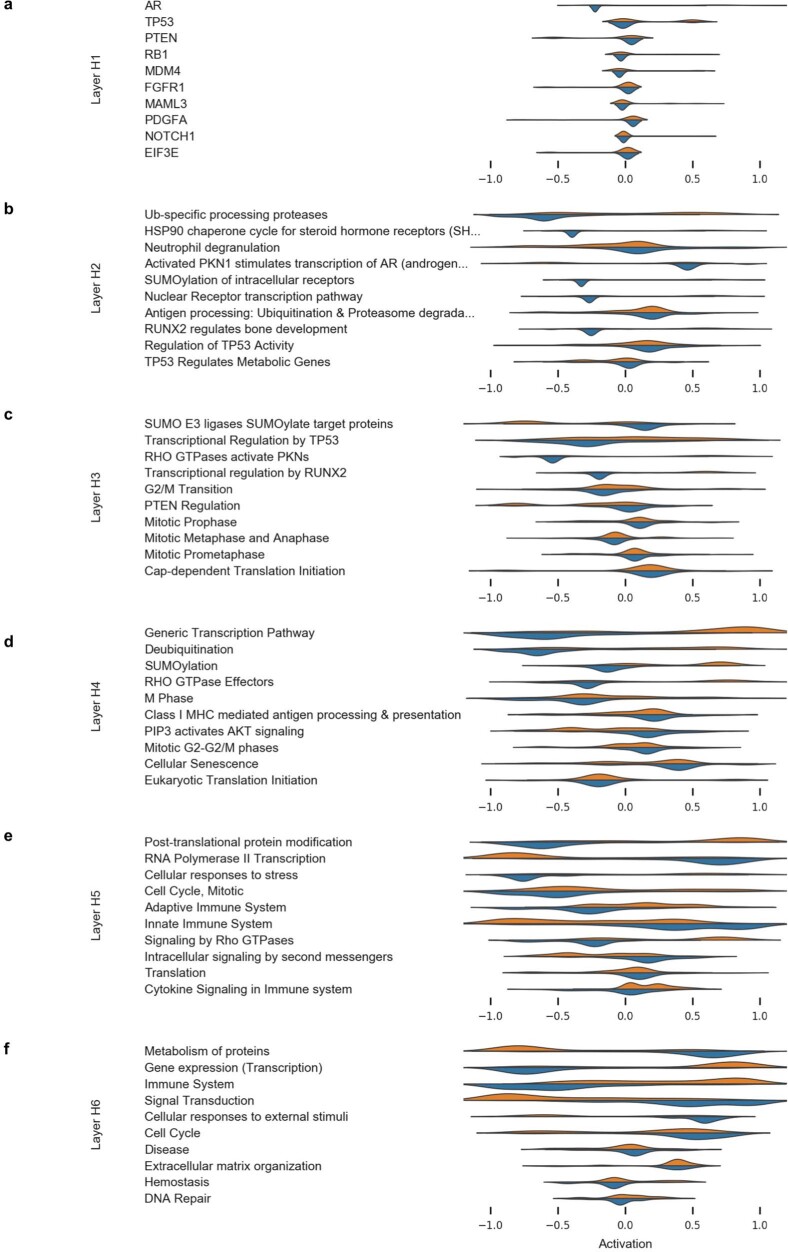
Activation distribution of important nodes in each layer. The activation distribution of top ranked nodes in each layer. Nodes in each layer are ordered based on their total importance score. The shown distribution is estimated using kernel density estimation to estimate the underlying distribution of node activations calculated for the testing set (*n* = 204). The current implementation of P-NET uses *tanh* activation function so the activation values are in the range −1 to 1. The figure shows better discrimination between sample classes (Primary- blue vs. Metastatic-orange) in higher layers compared to lower layers and in top ranked nodes compared to lower ranked ones. This shows that the total importance score of the nodes is manifested locally through the differential activation of nodes (nodes process different samples differently).

To evaluate the relative importance of specific genes contributing to the model prediction, we inspected the genes layer and used the DeepLIFT attribution method to obtain the total importance score of genes (Methods)^13^. Highly ranked genes included *AR*, *PTEN*, *RB1* and *TP53*, which are known prostate cancer drivers previously associated with metastatic disease^1,2,9,32^. In addition, alterations in less expected genes, such as *MDM4*, *FGFR1, NOTCH1*^33^ and *PDGFA*, strongly contributed to predictive performance (Extended Data Fig. 6, 7). To understand the behaviour of trained P-NET, we checked the activation of each node in the network, where activation here represents the signed outcome of a certain node given its inputs, and tested whether this activation changed with the change of the input sample class (primary versus metastatic) (Methods). We observed that the difference in the node activation was higher in higher layers and more concentrated in highly ranked nodes in each layer (Extended Data Fig. 8). For example, the activation distribution of the nodes of layer H3 was different when P-NET was given a primary sample compared with a resistant sample (Extended Data Fig. 8c). Thus, the interpretable architecture of P-NET can be interrogated to understand how the input information is transformed through layers and nodes, enabling further understanding of the state and importance of the involved biological entities.

Through evaluation of multiple layers in the P-NET trained model, we observed convergence in *TP53-*associated biology contributing to CRPC. Tracing the relevance of *TP53*-related pathways to the gene levels, roles for *TP53* and *MDM2* have been previously established in prostate cancer disease progression^32,34–40^, we also observed alterations in *MDM4* that contributed substantially to this network convergence. *MDM4* can inhibit wild-type *TP53* expression by binding to and masking the transcriptional activation domain^40^, although its role in prostate cancer treatment resistance is incompletely characterized^41^.

**Fig. 4:**
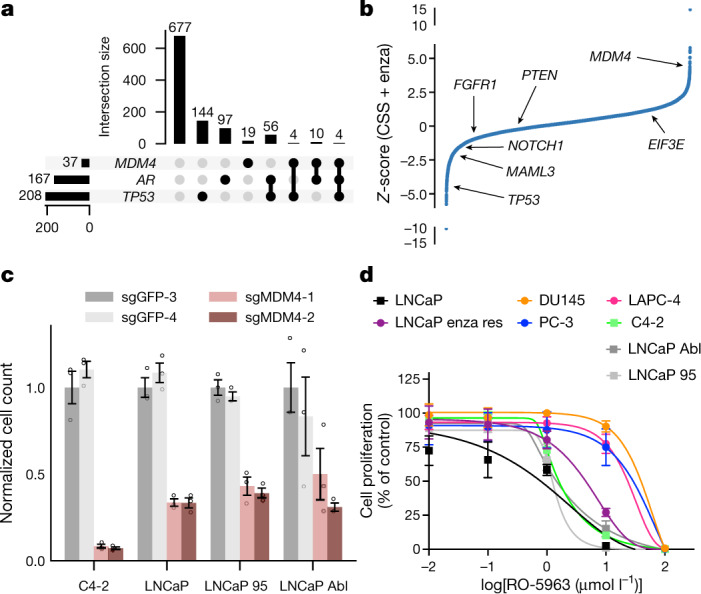
Clinical and functional evaluation of P-NET. **a**, Joint distribution of *AR*, *TP53* and *MDM4* alterations across 1,013 prostate cancer samples using an UpSetPlot^55^. A gene is defined as altered if it has a mutation, deep deletion or high amplification. **b**, Analysis of enzalutamide (enza)-resistant genes in LNCaP cells based on a genome-scale screen including 17,255 ORFs^42^. The relative enzalutamide resistance of each ORF (*x*-axis) is plotted as a *Z*-score (*y*-axis), with higher *Z*-scores representing more resistance (Supplementary Table 10). *MDM4* and other gene hits are highlighted on the graph, with *MDM4* scoring as the strongest hit among these genes. CSS, low androgen medium. **c**, Relative viability of C4-2, LNCaP, LNCaP Abl and LNCaP 95 cells after transduction of CRISPR–Cas9 and sgRNAs targeting *MDM4* (2 guides) or control GFP (2 guides). Data are mean ± s.e.m. of three replicates (the experiment was repeated three times with three replicates; Supplementary Data 1). **d**, Sensitivity of different prostate cancer cell lines to RO-5963. Relative viability is shown at each indicated dosage of RO5963. Data are mean ± s.d. of three replicates (the experiment was repeated three times; Supplementary Data 4). DU145, PC-3 and LAPC-4 are *TP53*-mutant prostate cancer cells; the other cells are *TP53* wild type.

**Extended Data Fig. 9:**
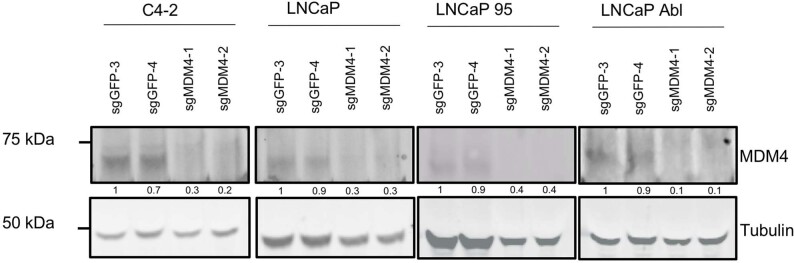
Immunoblot confirming *MDM4* gene deletion. Immunoblot confirming *MDM4* gene deletion in all cell lines used in Fig. 4-c. Tubulin is a loading control. Quantification of *MDM4* depletion is given under the *MDM4* blots. ImageStudioLite was used for quantification (Quantification numbers are included in Supplementary Data 3). The experiment was repeated 3 times with similar results.

We further studied the *MDM4* profile both in clinical samples and in functional models. High amplification of *MDM4* was more prevalent in resistant samples compared with primary samples (*χ*^2^ Yates correction = 40.8251, *P* < 0.00001). Alterations in *AR*, *TP53*, and *MDM4* genes are depicted in Fig. 4a. In a genome-wide gain-of-function preclinical screen using 17,255 open reading frames (ORFs) in LNCaP cells, *MDM4* overexpression was significantly associated with resistance to enzalutamide, a second-generation antiandrogen medication which is used for patients with CRPC^42^ (Fig. 4b). We then used CRISPR–Cas9 to target *MDM4* in multiple prostate cancer cell lines (Methods). Compared with a negative control, proliferation of prostate cancer cells was significantly reduced (*P* < 0.0001; *t*-test) (Fig. 4c; Supplementary Data 1) in response to *MDM4* depletion using two distinct single-guide RNAs (sgRNAs) (Extended Data Fig. 9, Supplementary Data 2). This indicated that selective therapeutic targeting of *MDM4* may be viable in patients with *TP53*-wild-type advanced prostate cancer. We thus sought to study the effect of inhibiting *MDM4* in prostate cell lines with mutant and wild-type *TP53*. Prostate cells with wild-type *TP53* were more sensitive to the *MDM4* selective inhibitor RO-5963 (which also inhibits MDM2) compared with *TP53*-mutant cell lines^43^ (Fig. 4d; Methods). Overall, convergence of p53 pathway dysregulation across multiple layers of the trained P-NET model identified specific vulnerabilities involving *MDM4*, which can be therapeutically targeted with MDM4-selective inhibition in a genomically stratified prostate cancer patient population.

## Discussion

Broadly, P-NET leveraged a biologically informed, rather than arbitrarily overparameterized, architecture for prediction. As a result, P-NET markedly reduced the number of parameters for learning, which led to enhanced interpretability. The sparse architecture in P-NET has better predictive performance when compared to other machine learning models, including dense networks, and may be applicable to other similar tasks. Application of P-NET to a molecular cohort of patients with prostate cancer demonstrated (1) model performance that may enable prediction of clinically aggressive disease in populations of patients with primary prostate cancer, and (2) convergent biological processes that contribute to a metastatic prostate cancer clinical phenotype that harbour novel therapeutic strategies in molecularly stratified populations.

Furthermore, P-NET provided a simple way for integrating multiple molecular features (for example, mutations, copy number variations and fusions, among others) weighted differently to reflect their importance in predicting the final outcome, which previously required different statistical approaches for each feature to enable cancer gene discovery^44,45^. Even more, P-NET provided a framework for encoding hierarchical prior knowledge using neural network languages and turning these hierarchies into a computational model that can be used both for prediction and for biological discovery in clinicogenomic contexts. Specifically, P-NET accurately predicted advanced prostate disease based on patients’ genomic profiles and had the ability to predict potential biochemical recurrence. Visualization of the architecture of P-NET enabled a multilevel view of the involved biological pathways and processes, which may guide researchers to develop hypotheses regarding the underlying biological processes involved in cancer progression and translate these discoveries into therapeutic opportunities. Specifically, P-NET rediscovered known genes implicated in CRPC, such as *AR*, *PTEN*, *TP53* and *RB1*. Moreover, P-NET identified *MDM4* as a relevant gene in this clinical context, which was experimentally validated and may inform use of *MDM4*-selective inhibitors for genomically stratified (*TP53*-wild-type) patients with metastatic prostate cancer.

Whereas P-NET provides a framework for outcome prediction and hypothesis generation, the model still requires tuning and training before being used. As with all deep learning models, the final trained model heavily depends on the hyperparameters used to train the model. In addition, P-NET encodes biological pathways inside the network in a hardcoded way, which makes the model dependent on the quality of the annotations used to build the model. Use of models that leverage other hardcoded biological priors (such as KEGG and Gene Ontology) or user-specified specific biological modules may further guide model development and functional evaluation. Finally, advances in computation may enable use of this approach in a patient-specific precision oncology schematic, paired with patient-specific model systems for directly comparable experimental assessments. Thus, the portability of this approach across different histological and clinical contexts requires further evaluation.

In conclusion, P-NET, a biologically informed deep neural network, accurately classified castration-resistant metastatic versus primary prostate cancers. Visualizing the trained model generated novel hypotheses for mechanisms of metastasis in prostate cancer and provided insights with direct potential for clinical translation in molecularly stratified prostate cancer patient populations. Biologically guided neural networks represent a novel approach to integrating cancer biology with machine learning by building mechanistic predictive models, providing a platform for biological discovery that may be broadly applicable across cancer prediction and discovery tasks.

## Methods

### P-NET design

We introduce P-NET, an artificial neural network with biologically informed, parsimonious architecture that accurately predicts metastasis in patients with prostate cancer on the basis of their genomic profiles. P-NET is a feedforward neural network with constraints on the nodes and edges. In P-NET, each node encodes some biological entity (for example, genes and pathways) and each edge represents a known relationship between the corresponding entities. The constraints on the nodes allow for better understanding of the state of different biological components. The constraints on the edges allow us to use a large number of nodes without increasing the number of edges, which leads to a smaller number of parameters compared to fully connected networks with the same number of nodes, and thus potentially fewer computations. The architecture was built using the Reactome pathway datasets^46^. The whole Reactome dataset was downloaded and processed to form a layered network of five layers of pathways, one layer of genes, and one layer for features. This sparse model had slightly over 71,000 weights with the number of nodes per layer distributed as shown in Extended Data Fig. 1e. A dense network with the same number of nodes would have more than 270 million weights with the first layer containing more than 94% of the weights. A hybrid model which contains a sparse layer followed by dense layers still contains over 14 million weights. The number of dense weights is calculated as *w*_*l*_ = *n*_*l*_ × (*n*_*l* – 1_ + 1), where *w*_*l*_ is the number of weights per layer *l* and *n*_*l*_ is the number of nodes of the same layer. Note that the P-NET model is not bound to a certain architecture, as the model architecture is automatically built by reading model specifications provided by the user via a gene matrix transposed file format (.gmt) file, and custom pathways, gene sets and modules with custom hierarchies can be provided by the user.

The meaning of the nodes, layers and connection of P-NET is encoded through a carefully engineered architecture and a set of restrictions on the connections of the network. The input layer is meant to represent features that can be measured and fed into the network. The second layer represents a set of genes of interest. The higher layers represent a hierarchy of pathways and biological processes that are manually curated. The first layer of P-NET is connected to the next layer via a set of one-to-one connections, and each node in the next layer is connected to exactly three nodes of the input layer representing mutations, copy number amplification and copy number deletions. This scheme results in a much smaller number of weights in the first layer compared with a fully connected network and the special pattern of the connection matrix results in more efficient training. The second layer is restricted to have connections reflecting the gene-pathway relationships as curated by the Reactome pathway dataset. The connections are encoded by a mask matrix *M* that is multiplied by the weights matrix *W* to zero-out all the connections that do not exist in the Reactome pathway dataset. For the next layers, a similar scheme is devised to control the connection between consecutive layers to reflect the real parent–child relationships that exist in the Reactome dataset. The output of each layer is calculated as *y* = *f* [(*M* *W)^*T*^*x* + **b**], where *f* is the activation function, *M* is the mask matrix, *W* is the weights matrix, *x* is the input matrix, **b** is the bias vector, and * is the Hadamard product (see Extended Data Fig. 1a–c). The activation of each node is kept into the range of [−1,1] by applying the tanh function $f={\tanh }={({\rm{e}}}^{2x}-1)/{({\rm{e}}}^{2x}+1)$ to the weighted inputs of the node. The activation of the outcome layers is calculated by the sigmoid function $\sigma =1/(1+{{\rm{e}}}^{-x})$.

To allow each layer to be useful by itself, we added a predictive layer with sigmoid activation after each hidden layer. P-NET has a smaller number of nodes per layer in the later layers compared to the first layers Extended Data Fig. 1e. Since it is more challenging to fit the data using a smaller number of weights in the later layers, we used a higher loss weight for later layer outcomes during the optimization process. The final prediction of the network was calculated by taking the average of all the layer outcomes, Extended Data Fig. 1d. The learning rate was initialized to be 0.001 and actively reduced after every 50 epochs to allow for smooth convergence. Since we have an unbalanced dataset, we weighted the classes differently to reduce the network bias toward one class based on the bias in the training set. The model was trained using Adam optimizer^47^ to reduce the binary cross-entropy loss functions $H=-\frac{1}{N}\Sigma {y}_{i}.{\rm{\log }}(p({y}_{i}))+(1-{y}_{i}).{\rm{\log }}(1-p({y}_{i}))$, where ${y}_{i}$ is the label for sample *i*, $p({y}_{i})$ is the probability that sample *i* has a metastatic cancer as calculated using the sigmoid function $\sigma$, and *N* is the total number of samples. Empirically we found that using adaptive learning rate besides Adam led to smoother convergence and improved prediction performance. We checked different gradient-based attribution methods to rank the features in all the layers, and we chose to use the DeepLIFT scheme as implemented in the DeepExplain library^13^.

DeepLIFT is a backpropagation-based attribution approach for assigning a sample-level importance score for each feature. In this work, we are interested in assigning scores for each node in each layer. Given a certain sample, a specific target *t*, and a set of layer nodes ${x}_{1}^{s},{x}_{2}^{s},{...,{x}}_{i}^{s},...,\,{x}_{{n}_{l}}^{s}$, where *n*_*l*_ is the number of nodes in a certain layer *l*, DeepLIFT calculates an importance score ${C}_{i}^{l,s}$ for each node on the basis of the difference in the target activation *t* – *t*_0_ such that the difference equals the aggregation of the calculated scores for all the nodes. That is, the difference in target activation is given by:

$$\triangle t=t-{t}_{0}$$

Which equals the sum of all node scores when fed by a certain sample $S$. That is,

$$\triangle t=\mathop{\sum }\limits_{i=1}^{{n}_{l}}{C}_{i}^{l,s}$$

We used the ‘Rescale rule’ of DeepLIFT as implemented by DeepExplain to calculate the sample-level importance of all nodes in all layers. Further details are available in ref. ^13^. To calculate the total node-level importance ${C}_{i}^{l}$ we aggregated the sample-level importance score scores over all the *n*_*s*_ testing-set samples.

$${C}_{i}^{l}={\rm{|}}\mathop{\sum }\limits_{i=1}^{{n}_{s}}{C}_{i}^{l,s}{\rm{|}}$$

Note that this is an absolute score (always positive) that measures the impact of a certain node on the outcome. The activation of the corresponding node *i*, however, could be positive or negative.

To reduce the bias introduced by over-annotation of certain nodes (nodes that are member of too many pathways), we adjusted the DeepLIFT scores using a graph informed function *f* that considers the connectivity of each node. The importance score ${C}_{i}^{l}$ is divided by the node degree ${d}_{i}^{l}$ if the node degree is larger than the mean of node degrees plus 5*σ* where *σ* is the standard deviation of node degrees.

$${d}_{i}^{l}={{fan}{\rm{\_}}{in}}_{i}^{l}+{{fan}{\rm{\_}}{out}}_{i}^{l}$$

$${{adjusted}{\rm{\_}}C}_{i}^{l}=f(x)=\{\begin{array}{c}\frac{{C}_{i}^{l}}{{d}_{i}^{l}},\,{d}_{i}^{l} > \mu +5\sigma \\ {C}_{i}^{l},\,{otherwise}\end{array}$$

### P-NET training and evaluation

To check the utility of the developed model, we trained P-NET to predict cancer state (primary/metastatic) of patients with prostate cancer on the basis of their genomic profiles. We used tumour or germline-matched whole-exome sequencing of 1,013 patients along with the corresponding somatic mutations and copy number alterations that were prepared using a unified computational pipeline for harmonized somatic alteration derivation^8^ (annotated in this study as the ‘Armenia et al.’ cohort). The mutations were aggregated on the gene level with focus on nonsynonymous mutations to align with prior work on mutational significance in prostate cancer whole-exome datasets, excluding silent, intron, 3′ untranslated region (UTR), 5′ UTR, RNA and long intergenic non-coding RNA (lincRNA) mutations. The copy number alterations for each gene were assigned on the basis of the called segment-level copy number emphasizing high gains and deep deletions and excluding single-copy amplification and deletions, as defined by GISTIC2.0 and generated from the source data type. For secondary analyses involving RNA data (fusions, expression), bulk whole transcriptomes from the subset of the Armenia et al. cohort, where such data were available, were secured from their source studies (*n* = 455 from TCGA, *n* = 204 from SU2C-PCF consortia) for uniform alignment and quantification of RNA sequences. Reads were downloaded as FASTQs from TCGA (ISB-CGC; https://isb-cgc.appspot.com/) and as CRAMs from SU2C (from Amazon S3 bucket, dbGaP accession code, phs000915.v2.p2) and then converted to FASTQs using samtools fastq. In cases where an SU2C sample had both transcriptome capture and polyA sequencing, transcriptome capture was used to optimize for fusion detection as the primary use of these data. Adapters were trimmed with cutadapt v2.2 and reads were aligned using STAR aligner v2.7.2b^48,49^. STAR-aligned bam files were passed into RSEM to generate gene-level transcript counts and transcript per million (TPM) quantifications using the GENCODE release 30 gene annotation lifted over to GRCh37. STAR chimeric junctions were supplied to STAR-Fusion v1.7.0 in kickstart mode to call fusions^50^. Fusion calls were filtered down to those that included genes classified as oncogene or fusion in the Cancer Gene Census^51^. To test model flexibility for RNA-based fusion inputs, as a secondary analysis we also developed P-NET models trained to predict cancer state incorporating fusions or different definitions of copy number states (Extended Data Fig. 3, 4).

The prediction performance was measured using the average AUC, the AUPRC and the F1 score. The corresponding measures were reported for the testing split and also for the cross-validation setup. The input data were divided into a testing set (10%) and a development set (90%). The development set was further divided into a validation set that has the same size as the testing set and the remaining samples are reserved for training. For the cross-validation experiments, the development dataset was divided into five folds stratified by the label classes to account for the bias in the dataset. The external validation results are produced by a model that is trained on the main dataset and tested on two independent external validation datasets. To mitigate the bias issue in the main dataset, we trained two models on two balanced subsamples drawn from the main dataset. The prediction scores of the two models are averaged to produce the final predictions on the two external validation datasets. The implementation of the proposed system along with the reproducible results are available on GitHub (https://github.com/marakeby/pnet_prostate_paper).

### Statistical analysis

The change in the area under the ROC curve between P-NET and other models is tested using DeLong test^52^. The *P*-values are corrected for multiple hypothesis testing using FDR. For other scores including AUPRC, accuracy, F1 and recall, bootstrapping statistical test with 2,000 sampling is used and the difference in score median was tested for significance. The resulting *P* value was corrected using the false-discovery rate (FDR) method. The AUC of five-fold cross-validation resulting from training and testing P-NET and dense models over multiple sample sizes is compared using a *t*-test of the means for the null hypothesis that two samples (P-NET scores and dense scores) have identical average (expected) values with the assumption that the populations have identical variances. The same test is applied to other scores including recall, precision, AUPRC, F1 and accuracy. For the survival analysis (Fig. 2d), a nonparametric log-rank test is used to compare estimates of the hazard functions of the two groups at each observed event time. A *t*-test of means is used to compare the reduction of prostate cancer cells proliferation in comparison to negative control in response to *MDM4* depletion. Chi-squared test with Yates correction is used to compare the expected and observed frequencies of *MDM4* high amplifications in two groups (patients with primary and metastatic tumours).

### Analysis of a genome-scale ORF screen

A genome-scale ORF screen was previously performed in LNCaP cells^42^. In brief, cells were infected with a pooled ORF library, subject to puromycin selection to isolate cells containing the respective ORFs, and then seeded in low androgen medium (CSS) with enzalutamide. The relative effect of each ORF on cell proliferation was determined after 25 days in culture and is represented as *Z*-scores. Raw results of the ORF screen were obtained from the Hwang et al. source study. We postulated that amplified genes identified by P-NET regulate oncogenic functions in metastatic CRPC. To validate this hypothesis, we analysed this previously published genome-scale ORF screen performed in LNCaP cells, which identified genes that, when overexpressed, promoted resistance to the *AR* inhibitor, enzalutamide^42^ (Fig. 4b). LNCaP cells are dependent on *AR* and treatment with enzalutamide attenuates cell proliferation. On the basis of this analysis, *MDM4* scored as a robust enzalutamide-resistant gene relative to other hits, including cell cycle regulators (*CDK4* and *CDK6*) or those with roles in FGF signalling (*FGFR2*, *FGFR3* and *FGF6*); these are two pathways implicated in driving resistance to anti-androgen therapies in clinical prostate cancers^27,53^.

### Sensitivity to RO-5963

LNCaP, LNCaP Abl, LNCaP 95, DU145, LAPC-4, LNCaP enzalutamide resistant, C4-2 and PC3 cells were seeded in 96-well plates at a density of 3,000 cells per well. After 24 h, cells were treated with increasing concentrations of RO-5963 for 4 days. Cell proliferation was determined using CellTiter-Glo assay. IC_50_ values were determined using GraphPad Prism. Data are represented as the mean ± s.d. of three replicates. The experiment was repeated three times (raw data and analysis files in Supplementary Data 4). All cell lines tested negative for mycoplasma contamination. Authentication was performed using STR profiles and/or obtained directly from ATCC for all publicly available cell lines.

### *MDM4* gene-depletion experiments

Blasticidin-resistant Cas9-positive prostate cancer cells were cultured in 150 μg ml^–1^ blasticidin (Thermo Fisher Scientific, NC9016621) for 72 h to enrich cells with optimal Cas9 activity. One million cells were seeded in parallel in 12-well plates and infected with lentiviruses expressing puromycin-resistant sgRNAs targeting *MDM4* or GFP control. Cells were then subjected to puromycin selection for 3 days and then the cells were counted using a Vi-Cell and seeded for a proliferation assay. 7 days later, cells were counted again with a Vi-Cell to assess viability, representing a total of 12 days. The target sequence against GFP was CACCGGCCACAAGTTCAGCGTGTCG (sgGFP). The target sequences against *MDM4* were AGATGTTGAACACTGAGCAG (sgMDM4-1) and CTCTCCTGGACAAATCAATC (sgMDM4-2).

### Immunoblotting

Cells were lysed using 2× sample buffer (62.5 mM Tris pH 6.8, 2% SDS, 10% glycerol, Coomassie dye) and freshly added 4% β-mercaptoethanol. Lysed cells were scraped, transferred into a 1.5 ml microcentrifuge tube, sonicated for 15 s and boiled at 95 °C for 10 min.

Proteins were resolved in NuPAGE 4–12% Bis-Tris Protein gels (Thermo Fisher Scientific) and run with NuPAGE MOPS SDS Running Buffer (Thermo Fisher Scientific, NP0001). Proteins were transferred to nitrocellulose membranes using an iBlot apparatus (Thermo Fisher Scientific). Membranes were blocked in Odyssey Blocking Buffer (LI-COR Biosciences, 927-70010) for 1 h at room temperature, and membranes were then cut and incubated in primary antibodies diluted in Odyssey Blocking Buffer at 4 °C overnight. The following morning, membranes were washed with phosphate-buffered saline with 0.1% Tween (PBST) and incubated with fluorescent anti-rabbit or anti-mouse secondary antibodies at a dilution of 1:5,000 (Thermo Fisher Scientific, NC9401842 (rabbit) and NC0046410 (mouse)) for 1 h at room temperature. Membranes were again washed with PBST and then imaged using an Odyssey Imaging System (LI-COR Biosciences). Primary antibodies used include *MDM4* (Abcam, ab16058) at a dilution of 1:500 and α-tubulin (Sigma, T9026) at a dilution of 1:1,000.

### Gene depletion of *MDM4* reduces prostate cancer cell viability

To determine how prostate cancer cells would respond to precision tools that target *MDM4* at the gene level, we used CRISPR-Cas9 and two sgRNAs targeting distinct sequences of *MDM4* in prostate cancer cell lines. Compared with a negative-control sgRNA (GFP), viability of 4 different prostate cancer cells was reduced by about 50–80% (Fig. 4c) in response to *MDM4* depletion (Extended Data Fig. 9) after 12 days in culture. Altogether, we concluded that *MDM4* regulates enzalutamide resistance, and that targeting *MDM4* through either chemical or genetic approaches significantly attenuated the viability of prostate cancer cell lines. Our observations indicate that antagonizing *MDM4* in metastatic CRPCs that harbour wild-type p53 is an attractive precision strategy. *MDM4* antibodies (A300-287A) and (ab16058) were used together for immunoblotting experiments done in Extended Data Fig. 9.

### Chemical inhibition of *MDM4* reduces prostate cancer cell viability

Given the proposed role of *MDM4* in driving enzalutamide resistance in prostate cancer cells, we sought to determine the response of prostate cancer cells to chemical inhibition of *MDM4*. We evaluated RO-5963, a small molecule *MDM2*/*4* dual inhibitor with the greatest selectivity towards *MDM4* in its class^43^. This drug has previously demonstrated robust efficacy against *MDM4* dependent cancer cell lines^54^. We evaluated the effects of increasing concentrations of RO-5963 on prostate cancer cell proliferation.

### Reporting summary

Further information on research design is available in the Nature Research Reporting Summary linked to this paper.

## Online content

Any methods, additional references, Nature Research reporting summaries, source data, extended data, supplementary information, acknowledgements, peer review information; details of author contributions and competing interests; and statements of data and code availability are available at 10.1038/s41586-021-03922-4.

## Supplementary information

Reporting Summary

Supplementary Tables 1–10 Supplementary Table 1 IDs of samples used in training. Supplementary Table 2 IDs of samples used in testing. Supplementary Table 3 IDs of samples used in validation. Supplementary Table 4 External validation (Robinson et al. (2017)). Supplementary Table 5 External validation (primary). Supplementary Table 6 FDR-adjusted table for comparing P-NET to other models. Supplementary Table 7 P-NET compared to other models. Supplementary Table 8 *P*-values of comparing P-NET to dense models when trained on different sample size. Supplementary Table 9 Survival data. Supplementary Table 10 functional ORF screen *z*-score list in all conditions from Hwang et al. (2019) used in Fig. 4

Supplementary Data 1 KD MDM4 proliferation raw data of 3 experiments in 4 cell lines in triplicates

Supplementary Data 2 MDM4 gene depletion experiments

Supplementary Data 3 Quantification of MDM4 of 3 western blot experiments

Supplementary Data 4 MDM4 inhibitor raw data, analysis files, figures and IC50

## Data Availability

All data used and generated from this study are deposited in 10.5281/zenodo.5163213. These datasets were derived from the following public domain resources^8,24,25,46^. The main dataset^8^ was downloaded from https://static-content.springer.com/esm/art%3A10.1038%2Fs41588-018-0078-z/MediaObjects/41588_2018_78_MOESM6_ESM.xlsx; https://static-content.springer.com/esm/art%3A10.1038%2Fs41588-018-0078-z/MediaObjects/41588_2018_78_MOESM4_ESM.txt; https://static-content.springer.com/esm/art%3A10.1038%2Fs41588-018-0078-z/MediaObjects/41588_2018_78_MOESM10_ESM.txt; https://static-content.springer.com/esm/art%3A10.1038%2Fs41588-018-0078-z/MediaObjects/41588_2018_78_MOESM10_ESM.txt; and https://static-content.springer.com/esm/art%3A10.1038%2Fs41588-018-0078-z/MediaObjects/41588_2018_78_MOESM5_ESM.xlsx. The external validation dataset^24,25^ was downloaded from https://met500.path.med.umich.edu/met500_download_datasets/somatic_v4.csv; https://static-content.springer.com/esm/art%3A10.1038%2Fnature20788/MediaObjects/41586_2017_BFnature20788_MOESM324_ESM.zip; and https://static-content.springer.com/esm/art%3A10.1038%2Fnature20788/MediaObjects/41586_2017_BFnature20788_MOESM325_ESM.zip.

## Code availability

Custom code was developed as part of the analysis reported here, and has been deposited on GitHub: https://github.com/marakeby/pnet_prostate_paper. The library names and versions used in the implementation are provided in https://github.com/marakeby/pnet_prostate_paper/blob/master/environment.yml. The final version of the code will be permanently available, after publication acceptance, on 10.5281/zenodo.5163855.
