# Supplementary material for: Biologically informed deep neural network for prostate cancer discovery
Source: Nature. 2021 Sep 22;598(7880):348–52. doi: 10.1038/s41586-021-03922-4 (PMC8514339; doi:10.1038/s41586-021-03922-4)
Supplement: Supplementary file 1 — Reporting Summary [file 41586_2021_3922_MOESM1_ESM.pdf]

nature research

Corresponding author(s): Eliezer Van Allen

Last updated by author(s): 08/05/2021

Reporting Summary

Nature Research wishes to improve the reproducibility of the work that we publish. This form provides structure for consistency and transparency in reporting. For further information on Nature Research policies, see our Editorial Policies and the Editorial Policy Checklist.

Statistics

For all statistical analyses, confirm that the following items are present in the figure legend, table legend, main text, or Methods section.

n/a	Confirmed	
☐	☒	The exact sample size (n) for each experimental group/condition, given as a discrete number and unit of measurement
☐	☒	A statement on whether measurements were taken from distinct samples or whether the same sample was measured repeatedly
☐	☒	The statistical test(s) used AND whether they are one- or two-sided<br>*Only common tests should be described solely by name; describe more complex techniques in the Methods section.*
☐	☒	A description of all covariates tested
☐	☒	A description of any assumptions or corrections, such as tests of normality and adjustment for multiple comparisons
☐	☒	A full description of the statistical parameters including central tendency (e.g. means) or other basic estimates (e.g. regression coefficient) AND variation (e.g. standard deviation) or associated estimates of uncertainty (e.g. confidence intervals)
☐	☒	For null hypothesis testing, the test statistic (e.g. F, t, r) with confidence intervals, effect sizes, degrees of freedom and P value noted<br>*Give P values as exact values whenever suitable.*
☒	☐	For Bayesian analysis, information on the choice of priors and Markov chain Monte Carlo settings
☒	☐	For hierarchical and complex designs, identification of the appropriate level for tests and full reporting of outcomes
☒	☐	Estimates of effect sizes (e.g. Cohen's d, Pearson's r), indicating how they were calculated

Our web collection on statistics for biologists contains articles on many of the points above.

Software and code

Policy information about availability of computer code

Data collection	Data used in the study is available in the public domain. No special software was used to collect the data.
Data analysis	A custom code was developed as part of the analysis reported here. The full code is deposited on the code sharing site GitHub and the link is provided in the Methods section of the submitted paper (https://github.com/marakeby/pnet_prostate_paper)<br>The library names and versions used in the implementation are provided in https://github.com/marakeby/pnet_prostate_paper/blob/master/environment.yml<br>ImageStudioLite was used for quantification of MDM4 depletion<br>GraphPad Prism 9.1.2 was used to determine IC50 values

For manuscripts utilizing custom algorithms or software that are central to the research but not yet described in published literature, software must be made available to editors and reviewers. We strongly encourage code deposition in a community repository (e.g. GitHub). See the Nature Research guidelines for submitting code & software for further information.

Data

Policy information about availability of data

All manuscripts must include a data availability statement. This statement should provide the following information, where applicable:

- Accession codes, unique identifiers, or web links for publicly available datasets
- A list of figures that have associated raw data
- A description of any restrictions on data availability

All data used and generated from this study are deposited in https://doi.org/10.5281/zenodo.5163213
These datasets were derived from the following public domain resources:
Armenia J, Wankowicz SAM, Liu D, Gao J, Kundra R, Reznik E, et al. The long tail of oncogenic drivers in prostate cancer. Nat Genet. 2018;50: 645–651. DOI:

10.1038/s41588-018-0078-z
Fraser M, Sabelnykova VY, Yamaguchi TN, Heisler LE, Livingstone J, Huang V, et al. Genomic hallmarks of localized, non-indolent prostate cancer. Nature. 2017;541: 359–364. https://doi.org/10.1038/nature20788
Robinson DR, Wu Y-M, Lonigro RJ, Vats P, Cobain E, Everett J, et al. Integrative clinical genomics of metastatic cancer. Nature. 2017;548: 297–303. https://doi.org/10.1038/nature23306
Fabregat A, Jupe S, Matthews L, Sidiropoulos K, Gillespie M, Garapati P, et al. The Reactome Pathway Knowledgebase. Nucleic Acids Res. 2018;46: D649–D655. DOI: 10.1093/nar/gkv1351

Field-specific reporting

Please select the one below that is the best fit for your research. If you are not sure, read the appropriate sections before making your selection.

☒ Life sciences ☐ Behavioural & social sciences ☐ Ecological, evolutionary & environmental sciences

For a reference copy of the document with all sections, see nature.com/documents/nr-reporting-summary-flat.pdf

Life sciences study design

All studies must disclose on these points even when the disclosure is negative.

Sample size	Typical sample size and power calculations do not apply to non-linear machine learning models. Machine learning methodologies generally improve as sample sizes increase, which makes prospective power analyses in these contexts difficult to interpret given the nonlinearity of the underlying mathematical framework relative to the parametric approaches leveraged for power calculations. We explicitly studied the effect of the training sample size on the computational performance of the developed model in predicting clinical outcomes in unseen dataset and compared this to other machine learning models as well.
Data exclusions	no exclusions
Replication	All the analyses reported here are tested for reproducibility. Best practices of machine learning development are followed. Random seed is set for all experiments. Source code for reproducing the results are deposited on GitHub. Machine learning training and testing process is repeated 5 times in a randomized 5 fold cross-validation setup. Knock down experiments are repeated 3 times with 3 replicates in each experiment. Drug treatment experiments are repeated 3 times.
Randomization	Best practices for randomizing samples for machine learning model development were followed. Samples were randomly assigned to training, testing, and validation groups. The performance metrics of all machine learning models are reported and compared for testing group. The experiments are repeated in a randomized 5 cross-validation setup and the metrics are compared as well.
Blinding	Investigators were not blinded. Blinding during data collection was not needed because the data is collected from the public domain.<br>The developed machine learning models however were blinded to part of the data (the testing and external validation sets) to evaluate their computational performance after being trained on the training set.<br>Blinding is also not needed for the interpretation since the results are quantitative and did not require subjective judgment or interpretation.

Reporting for specific materials, systems and methods

We require information from authors about some types of materials, experimental systems and methods used in many studies. Here, indicate whether each material, system or method listed is relevant to your study. If you are not sure if a list item applies to your research, read the appropriate section before selecting a response.

Materials & experimental systems

n/a	Involved in the study	
☐	☒	Antibodies
☐	☒	Eukaryotic cell lines
☒	☐	Palaeontology and archaeology
☒	☐	Animals and other organisms
☒	☐	Human research participants
☒	☐	Clinical data
☒	☐	Dual use research of concern

Methods

n/a	Involved in the study	
☒	☐	ChIP-seq
☒	☐	Flow cytometry
☒	☐	MRI-based neuroimaging

Antibodies

Antibodies used	MDM4 (Thermo Fisher Scientific, A300287A, Abcam ab16058) , Alpha Tubulin (DM1A, Sigma T9026)
Validation	These antibodies have been examined in a previous study (Howard TP, et al. “MDM2 and MDM4 are Therapeutic Vulnerabilities in Malignant Rhabdoid Tumors”. Cancer Research 2019.). In the cited study, the current work as well as in unpublished experiments, we used various forms of gene knockdown (RNAi) or knock-out (CRISPR-Cas9) experiments to confirm the antibodies recognize the

appropriate target protein in multiple cell lines. The additional proteins tested are known interactions of the TP53 pathway that regulate expression of one another, we we observed the anticipate changes of these as "positive control" phenotypes.

Eukaryotic cell lines

Policy information about cell lines

Cell line source(s)	C4-2, DU145, PC3 (ATCC)<br>LAPC4, LNCaP95, LNCaP Abl, and LNCaP enz-resisant are used from published sources<br>LAPC4, (Ginevra/Levi's lab. https://web.expasy.org/cellosaurus/CVCL_4744)<br>LNCaP95, LNCaP Abl (Brown/Freedman's group https://pubmed.ncbi.nlm.nih.gov/10496349 ,https://pubmed.ncbi.nlm.nih.gov/19117982)<br>LNCaP enz-resisant (https://pubmed.ncbi.nlm.nih.gov/27036029/)
Authentication	Authentication performed using STR profiles and/or obtained directly from ATCC for all publicly available cell lines. For published cell lines, please contact the original owners of the cell lines.
Mycoplasma contamination	All cell lines were tested every 3-6 months MycoAlert (https://www.promega.com/resources/pubhub/applications-notes/detecting-mycoplasma-using-the-mycoalert-kit-on-the-glomax-2020/). Each cell line had been aliquoted post testing and frozen in liquid nitrogen. Cells are only thawed for experimentation and cultured up to 2 Mos. Results were negative for mycoplasma contamination.
Commonly misidentified lines<br>(See ICLAC register)	Cell lines used are NOT included on the commonly misidentified lines list.
