# Supplementary material for: Biologically informed deep neural network for prostate cancer discovery
Source: Nature. 2021 Sep 22;598(7880):348–52. doi: 10.1038/s41586-021-03922-4 (PMC8514339; doi:10.1038/s41586-021-03922-4)
Supplement: Supplementary file 4 — MDM4 gene depletion experiments [file 41586_2021_3922_MOESM4_ESM.pptx]

## Slide 1
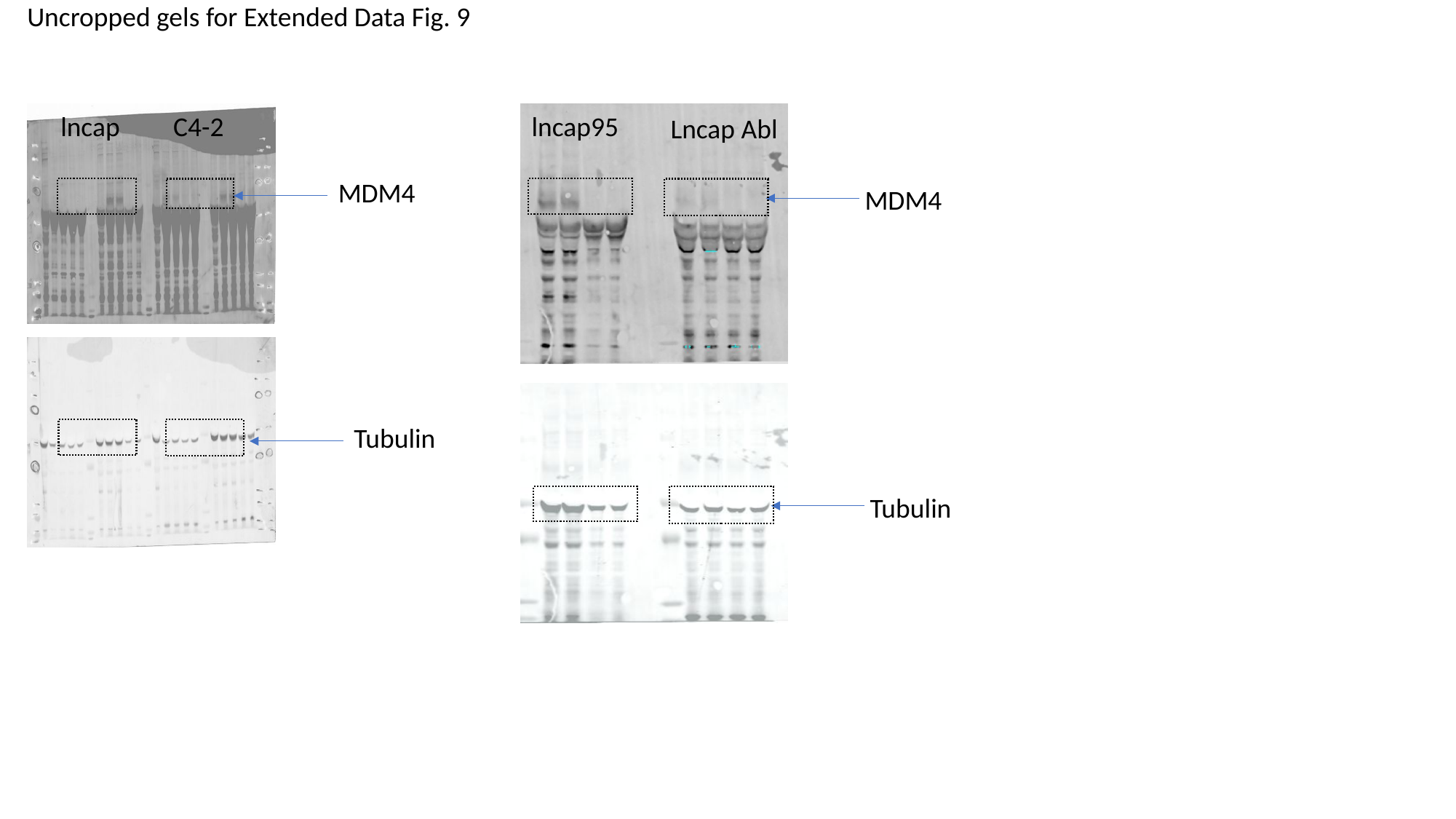

Uncropped gels for Extended Data Fig. 9
lncap
C4-2
lncap95
Lncap Abl
MDM4
MDM4
Tubulin
Tubulin

## Slide 2
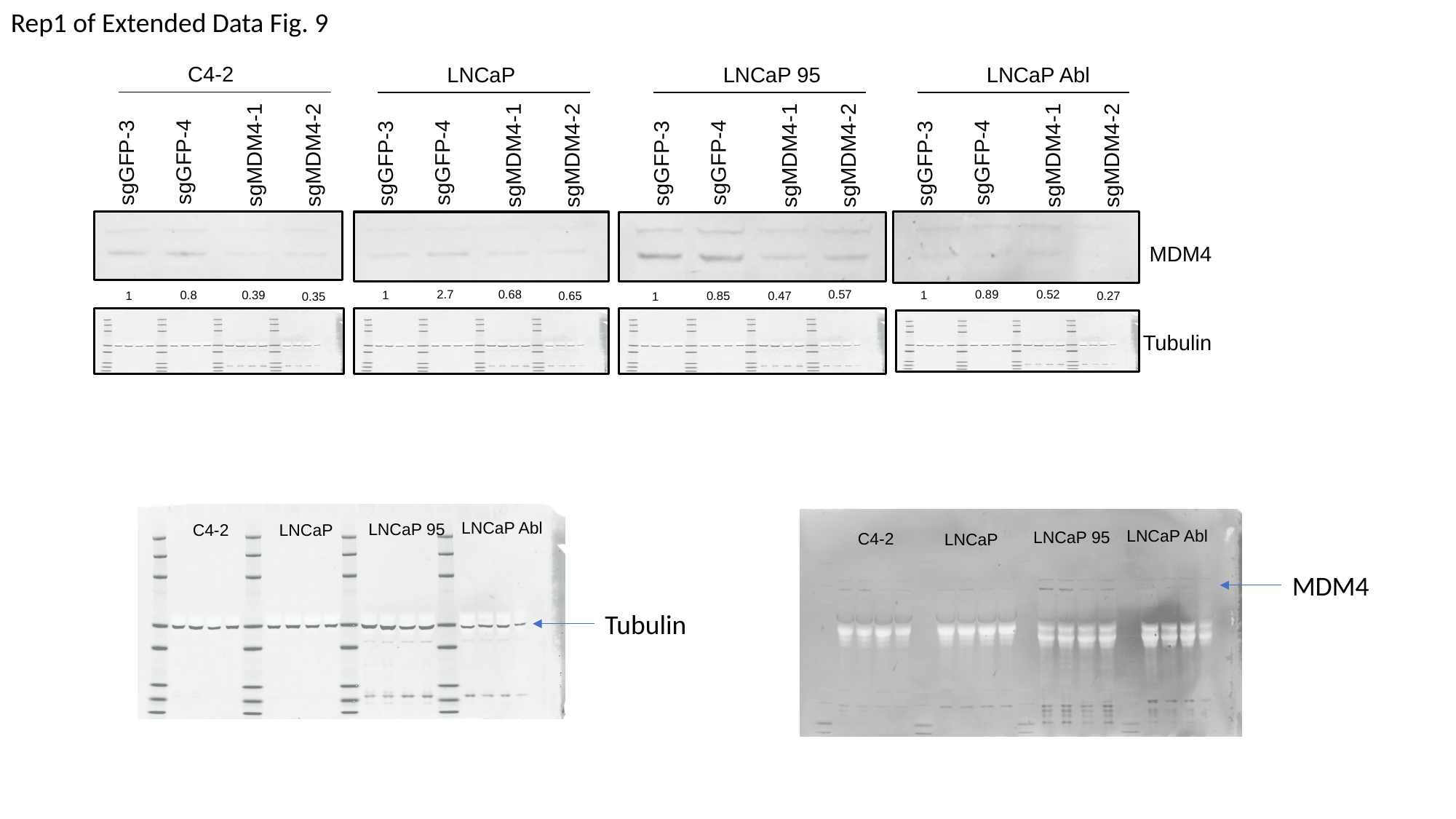

Rep1 of Extended Data Fig. 9
C4-2
LNCaP
LNCaP Abl
LNCaP 95
sgMDM4-1
sgMDM4-2
sgMDM4-1
sgMDM4-2
sgMDM4-1
sgMDM4-2
sgMDM4-1
sgMDM4-2
sgGFP-4
sgGFP-4
sgGFP-4
sgGFP-4
sgGFP-3
sgGFP-3
sgGFP-3
sgGFP-3
MDM4
0.57
2.7
0.68
0.89
0.52
0.8
0.39
1
1
1
0.85
0.47
0.65
0.27
1
0.35
Tubulin
LNCaP Abl
LNCaP 95
C4-2
LNCaP
LNCaP Abl
LNCaP 95
C4-2
LNCaP
MDM4
Tubulin

## Slide 3
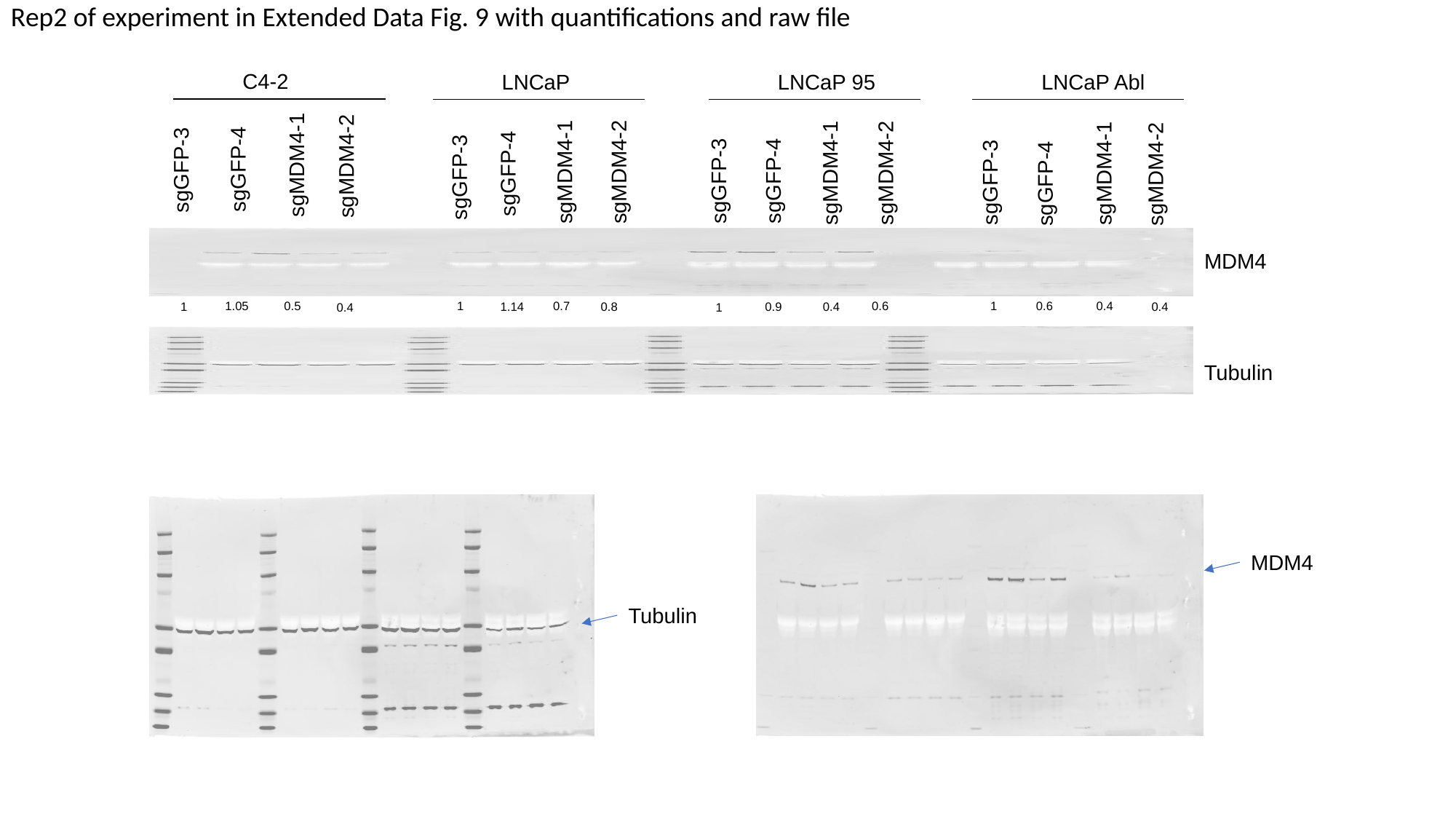

Rep2 of experiment in Extended Data Fig. 9 with quantifications and raw file
C4-2
LNCaP
LNCaP Abl
LNCaP 95
sgMDM4-1
sgMDM4-2
sgGFP-4
sgGFP-3
sgMDM4-2
sgMDM4-1
sgMDM4-1
sgMDM4-2
sgGFP-4
sgMDM4-1
sgMDM4-2
sgGFP-3
sgGFP-4
sgGFP-3
sgGFP-3
sgGFP-4
MDM4
0.6
0.7
0.6
0.4
1.05
0.5
1
1
1
1.14
0.9
0.4
0.8
0.4
1
0.4
Tubulin
MDM4
Tubulin

## Slide 4
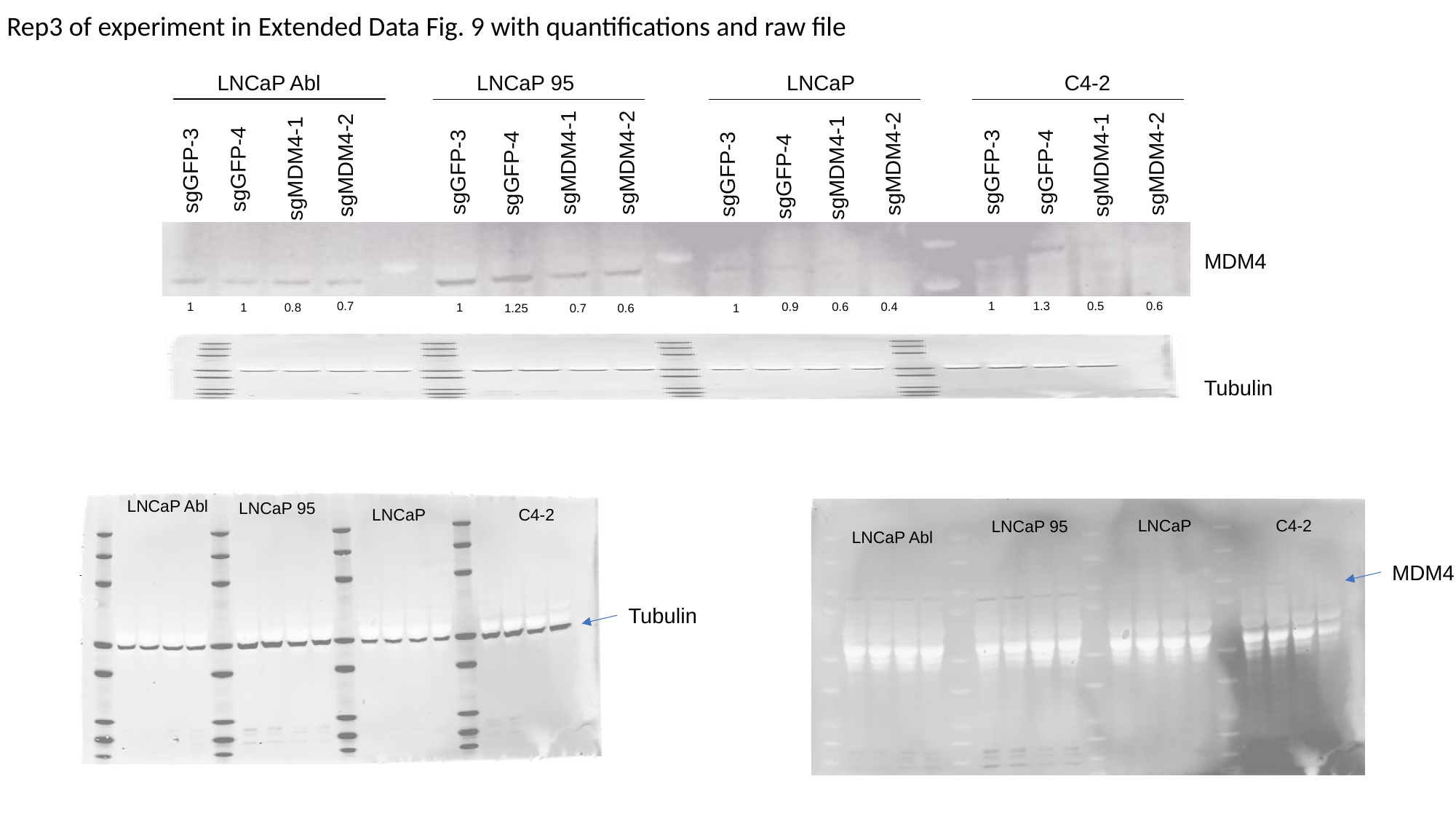

Rep3 of experiment in Extended Data Fig. 9 with quantifications and raw file
LNCaP Abl
LNCaP 95
LNCaP
C4-2
sgMDM4-1
sgMDM4-2
sgMDM4-2
sgMDM4-2
sgMDM4-1
sgMDM4-2
sgMDM4-1
sgMDM4-1
sgGFP-4
sgGFP-3
sgGFP-3
sgGFP-3
sgGFP-4
sgGFP-4
sgGFP-3
sgGFP-4
MDM4
0.7
0.5
0.6
1.3
1
1
0.6
0.4
0.9
0.8
1
1
1.25
1
0.7
0.6
Tubulin
LNCaP Abl
LNCaP 95
LNCaP
C4-2
LNCaP
C4-2
LNCaP 95
LNCaP Abl
MDM4
Tubulin
